# Influence of Processing and Digestion on the Stability, Bioac-Cessibility and Bioactivity of Food Polyphenols

**DOI:** 10.3390/foods12040851

**Published:** 2023-02-16

**Authors:** Angela Conte, Serena Martini, Davide Tagliazucchi

**Affiliations:** Department of Life Science, University of Modena and Reggio Emilia, Via Amendola 2, 42100 Reggio Emilia, Italy

In part, the role of polyphenols, as partially responsible components, for the protective effects of a fruit and vegetable-rich diet is an increasingly important area of human nutrition research.

Plant derived polyphenols are considered bioactive dietary compounds with protective effects against most chronic diseases, including cardiovascular diseases, cancer, and diabetes. Both epidemiological and clinical evidence suggest that diets rich in polyphenols can reduce the risk of developing these age-related chronic diseases. The determination of bioactive compounds exclusively in foodstuff is not enough to predict its potential effects in vivo. The potential of bioactive compounds to exert their health effect depends on food matrix release, food processing, changes during digestion, absorption, and cellular metabolism. Furthermore, the concentration of phytochemicals present in the target tissues in an active form, may depend on the binding to plasma proteins, on the metabolism in the liver, and on the processes of excretion for which it is also necessary to evaluate the pharmacokinetic aspects and the metabolic interactions. The bioactivity of polyphenols therefore depends on their bioaccessibility and bioavailability. The study of how all these different elements influence the bioaccessibility and bioactivity of polyphenols is also a fundamental support for the food industry which uses antioxidants and pigments as valid additives. The stability of polyphenols can be affected by their solubility, light, pH, oxygen, and temperature of the environment. They can be transformed into metabolites with different activity, may be retained inside epithelial cells during their transport across the intestinal barrier, or they can be destroyed during gastrointestinal digestion, resulting in lower bioaccessibility and bioactivity. Once in the colon, polyphenols are metabolized by the gastrointestinal microbiota to a diversity of phenolic and aromatic catabolites, which can be readily absorbed across the intestinal barrier into the bloodstream. Information on the metabolism of phenolic compounds during passage through the gastrointestinal tract is important to understand how much of their biological effects can be modified by the metabolism of intestinal microbiota and/or cells. Often after ingestion, dietary (poly)phenols appear in the circulatory system not as the parent compounds, but as their metabolites. Substantial quantities of both the parent compounds and their metabolites pass to the colon where they are metabolized and further modified by the action of the local microbiota, giving rise principally to a small phenolic acid and aromatic catabolites that are absorbed into the circulatory system. Profiling of metabolic and excretive properties is useful to understand the in vivo behavior and the mechanism of action of dietary phytochemicals. The nature of the food processing operations used also impacts the bioaccessibility and bioactivity of phytochemicals in complex ways. Most fruits and vegetables are not usually consumed raw, and they go through industrial or domestic processing steps (e.g., storage, cooling, heating, drying, fermentation, etc.) to make them available for human or animal consumption. Some specific goals include: the inactivation of pathogens or contaminating microorganisms to improve the shelf life of ingredients and products; the increase in the bioavailability of otherwise inaccessible nutrients; the variety of flavor, texture, or aroma of certain foods; the improvement of the nutritional profile. Domestic and industrial processing affects phenolic compounds content, antioxidant capacity, bioaccessibility, and bioavailability in different ways. For instance, thermal processing can decrease the bioavailability of phytochemicals by promoting their chemical degradation, or it can increase their bioavailability by enhancing their release from the food matrix. Combinations of thermal processing with other techniques, such as microwave, ultrasound, or vacuum methods, have been shown to reduce the negative impacts of heating on phytochemicals. Non-thermal technologies such as freeze-drying, high-pressure processing, pulsed electric field, and bioprocessing have also been shown to reduce the degradation and enhance the bioaccessibility of phytochemicals in food products.

The effect of cooking on phenolic compounds stability is dependent on a fine balance between their loss or their degradation, their reaction with food matrix components and the increased extractability due to the matrix softening effect. This equilibrium strongly relies upon the cooking conditions (such as time, temperature, and cooking medium), the food matrix, and the phenolic structure. The different cooking procedures may have a pivotal impact on the bioaccessibility of phenolic compounds.

Knowledge of bioaccessibility and bioavailability is therefore essential to determine the nutritional quality of a nutrient or bioactive compound both in terms of quantities necessary to meet nutritional requirements and to fine-tune the development of functional foods. This Special Issue “Influence of Processing and Digestion on the Stability, Bioaccessibility and Bioactivity of Food Polyphenols” was launched to highlight recent and important research addressing interesting topics in this important area of nutrition.

Seke et al. [1] presented interesting results on the effectiveness of alginate/psyllium mucilage beads in improving stability and in vitro anthocyanin release. During simulated gastrointestinal digestion, the anthocyanins are highly unstable with low bioaccessibility. The low bioaccessibility of anthocyanins can hinder their functionality in the body. Indeed, the efficiency of anthocyanins depends on their integrity and bioaccessibility. The action of anthocyanins and their potential therapeutic benefits are limited by their instability during food preparation, circulation, or in the gastrointestinal tract. They degrade quickly due to rapid oxidation, which could hinder the effectiveness of using these polyphenols in the pharmaceutical and nutraceutical industries. A higher bioaccessibility of anthocyanins can be obtained with microencapsulation techniques that protect the bioactive compounds from adverse conditions during gastrointestinal digestion.

Cele et al. [2] investigated the fate and bioaccessibility of total phenolic compound, carotenoid components, antioxidant activities, and lactic acid bacteria survival in fermented mango juice obtained from different cultivars after exposure to in vitro gastrointestinal digestion. The digestion of lactic acid bacteria-fermented mango juices resulted in the depletion of bioaccessible and bioavailable phenolic compounds and the reduction of the antioxidant activities of fermented mango juices. Digestion of fermented mango juices had no significant reduction on the surviving lactic acid bacteria population, suggesting that lactic acid bacteria-fermented mango juices may have the potential to function as probiotics in the gut in addition to their antioxidative properties. The bioactive compounds and their metabolites in juices have been proven to function as prebiotics to promote probiotics proliferation. Therefore, fruit juice fermented with probiotics may be an effective strategy to meet market requirements and has the potential to be a nutritious probiotics beverage with biofunctions suitable for consumption by a wider range of individuals.

Soukup et al. [3] investigated the metabolism of daidzein and genistein using pure cultures of Eggerthellaceae and Coriobacteriaceae strains. This study led to the first description of the human gut bacterial strains ‘Hugonella massiliensis’ DSM 101782T and Senegalimassilia faecalis KGMB 04484T as capable of metabolizing daidzein and genistein. Isoflavones, especially daidzein, are highly bioavailable compared to most other (poly)phenols and isoflavone metabolites, and are being absorbed into the circulatory system from both the small and large intestine. For isoflavones, the endogenous transformation in humans is well known, and they can be metabolized by endogenous phase I and phase II enzymes and by gut bacteria, thus leading to the modulation of their bioactivity. High inter-individual variations in microbial metabolism are observed in humans. For example, only about one-third of the human population can convert daidzein to equol, an important metabolite, showing the highest affinity to estrogenic receptors among all known isoflavones; there is still limited knowledge of bacteria that are involved in this conversion.

Vesely et al. [4] described the biotransformation of selected 2-arylbenzofurans (mulberrofuran Y, mulberrofuran G, moracin C) by human microbiota and their cellular uptake through an intestinal in vitro model. 2-Arylbenzofurans have a stilbenoid-related structure and exhibit bioactive effects with potential applications in human nutrition and medicine. In this study, significant differences in the metabolism of 2-arylbenzofurans were found. Bacteria did not produce any metabolites from the three compounds investigated. Moreover, the results clearly indicated a difference in the sulfation and glucuronidation processes when investigating the permeability by intestinal cells. The compounds differed in the degree of their conversion in the intestinal model, providing important information on the initial bioavailability of 2-arylbenzofurans, which significantly influences their resulting bio-efficacy. Mulberrofuran G has antimicrobial effects and acts as a cyclooxygenase and lipoxygenase inhibitor. Mulberrofuran Y and moracin C have cytotoxic activity and inhibit the production of nitric oxide. Dietary intake of 2-arylbenzofurans can result in potential benefits, especially for chronical inflammatory diseases and tumor prevention.

Zeng et al. [5] have developed and validated an LC-MS/MS method for the simultaneous determination of ten flavonoid metabolites derived from naringin in rat urine. Thanks to its favorable bioactivities, naringin has been involved in numerous metabolism and excretion studies. Naringin is extensively hydrolyzed to naringenin, which can undergo a series of dehydrogenation, hydroxylation, methylation, glucuronidation, and sulfation reactions, resulting in a large amount of different metabolites. Although the metabolite profile of naringin is clear, quantitative studies have mostly focused on free naringin and naringenin obtained after the co-incubation with glucuronidase and/or sulfatase, resulting in the loss of information concerning the excretion profile of original metabolites, especially conjugates such as glucuronides and sulfates. In fact, there are few reports on the development and validation of methods for the simultaneous determination of multiple original metabolites derived from naringin in biological samples so far. With this method, ten analytes in rat urine could be simultaneously quantified within a short chromatographic elution time (11 min). The obtained results suggest that there exists a visible individual difference and low urinary recovery of flavonoid metabolites in the excretion of naringin and provide reference for studies concerning the excretion of other flavonoids.

Cattivelli et al. [6] assessed the effect of the four different and most common cooking methods on the stability, bioaccessibility and antioxidant activity of phenolic compounds from two onion varieties (yellow-skinned and red-skinned onion) after cooking and in vitro gastro-intestinal digestion. This study demonstrated that cooking treatments may modulate the release and bioaccessibility of onion phenolic compounds. All the treatments resulted in a decrease in the phenolic compounds content respect to the raw samples with the only exception found for fried yellow-skinned onions. After in vitro digestion, baking for both the onion varieties and grilling for red-skinned onions significantly increased the bioaccessibility of onion phenolic compounds. Baking was the thermal treatment that provided the highest amount of phenolic compounds for both the onion varieties. The results of this study suggest how baking and grilling are the recommended cooking methods, not only for the healthy lack of use of cooking oils or fats but also for the evidence of the proved bioaccessibility characteristics of onion health-promoting phenolic compounds (e.g., flavonols).

In their review Bertelli et al. [7] have deepened some fundamental aspects linked to the role of polyphenols in maintaining health, such as the limits of bioavailability and the metabolic interactions of this large class of secondary metabolites, the question of the poor absorption of polyphenols, and the role of phytocomplexes in understanding the strengths and the limits of in vitro studies. This knowledge provides practical hints to better address the investigation on the subject and the transfer to clinical trial and use in medicine. Polyphenols are natural compounds synthesized exclusively by plants, with different biological activities related to the ability to modulate oxidative and inflammatory stress, alter the digestion of macronutrients, and exert prebiotic-type effects on the intestinal microbiota. These compounds are widely distributed in foods of plant origin, and therefore in the human diet, and have reached an important position thanks to the significant evidence of a negative correlation of their consumption with tumors, diabetes, and cardiovascular diseases. Both epidemiological and clinical evidence suggest that diets rich in polyphenols may reduce the risk of several age-related chronic diseases.

About 200 relevant and recent articles on specific topics such as polyphenols bioavailability, polyphenols matrix effect, food matrix effect, and polyphenols-cytochrome interaction were considered in this review, while the most incisive and recent literature on chemical and biological properties has been extensively reviewed, looking at in vitro, in vivo, and clinical studies.

Concluding the presentation of this Special Issue, we would like to thank the research teams for their contributions to this Special Issue, which have provided novel information on such a complex topic as the bioaccessibility and bioactivity of polyphenols. These contributions could be useful for the development of more healthy food products, as well as for promoting further research in this important area.
